# Causal effects of genetically determined blood metabolites on multiple myeloma: a Mendelian randomization study

**DOI:** 10.1038/s41598-023-45801-0

**Published:** 2023-11-01

**Authors:** Jialin Ren, Min Wu

**Affiliations:** 1 Institute of Translational Medicine, Medical College, Yangzhou University, Yangzhou, 225009 China; 2 Jiangsu Key Laboratory of Integrated Traditional Chinese and Western Medicine for Prevention and Treatment of Senile Diseases, Yangzhou University, Yangzhou, 225009 China

**Keywords:** Molecular biology, Haematological diseases

## Abstract

Previous studies have shown that metabolites play an important role in phenotypic regulation. However, the causal relationship between metabolites and multiple myeloma has not been adequately investigated. Here, we attempt to explore the causal effects of genetically determined blood metabolites on multiple myeloma. The large-scale public blood metabolites and multiple myeloma datasets from independently published genome-wide association studies (GWAS) were used to explore the causal relationship between each genetically determined blood metabolite and multiple myeloma through inverse variance weighted (IVW), weighted median, MR-Egger and mode-based estimation methods. Sensitivity tests were performed to evaluate the stability and reliability of the results by MR-Egger regression and leave-one-out methods. Metabolic pathway analysis was further explored using filtered data. Statistical analyses were all performed in R. Among 452 metabolites, ten known metabolites and three unknown metabolites had significant causal relationship with multiple myeloma (*P* < 0.05). Four known metabolites, 3-methyl-2-oxovalenate, oxidized bilirubin, isovalerylcarnitine and glutamine carnitine, reached statistical significance in IVW models. Metabolic pathways analysis identified four significant pathways. The occurrence of multiple myeloma may have a causal relationship with these four metabolites, and there are four metabolic pathways that are also related to the occurrence of multiple myeloma. This can provide new ideas for exploring early screening and treatment of multiple myeloma.

## Introduction

Multiple myeloma (MM) is a hematological malignancy characterized by malignant proliferation of plasma cells in the bone marrow, accompanied by the secretion of monoclonal immunoglobulins. The disease accounts for 10% of all hematologic malignancies^1^. Despite substantial improvements in multiple myeloma therapies in the past 10–15 years, the 5-year relative survival rate is 55.6%^2^ and the median overall survival (OS) has improved to over 8 years^3^. But in 15–20% of patients the aggressive course of MM leads to death within the first 3 years from diagnosis^4^. Traditional chemotherapy is highly resistant and relapsed, and the advent of newer drugs has prolonged survival, but overall multiple myeloma remains incurable^5^. Therefore, it is important to investigate new targets for screening, prevention and treatment of multiple myeloma.

Metabolites in the blood can, to some extent, reflect an individual's genetic makeup and can therefore be used to predict or influence the onset and progression of disease^6^. Common genetic metabotypes play a role as discriminatory cofactors in the aetiology of common multifactorial diseases. Interacting with environmental factors such as diet or lifestyle, these metabotypes may influence an individual's susceptibility to certain phenotypes^7^. Genetic variants in metabolism-related genes that lead to specific and distinct metabolic phenotypes, which we call 'genetically determined metabotypes'^8^. Currently, genome-wide association studies (GWAS) have identified a number of metabolite-associated loci in adult human blood and/or urine samples that have been shown to be associated with the development and prognosis of cardiovascular^9,10^, endocrine^11^, gastrointestinal^12^, respiratory^13^ and oncological^14^ diseases. But few studies have focused on the relationship between blood metabolites and multiple myeloma. Mendelian randomization (MR) is a powerful method of epidemiologic research that essentially uses genetic variation as an instrumental variables (IVs) to identify causal relationships between risk factors and disease^15^. Genome-wide association studies (mGWAS) are a metabolomics-based approach to understanding disease-associated genetic variation by identifying genetic trait loci for metabolites^16^. Using this approach to investigate the causal relationship between blood metabolites and multiple myeloma may provide insight into multiple myeloma and new ideas for early detection and treatment of multiple myeloma.

In summary, this study combines metabolomics and genomics, through Mendelian randomization analysis, using large-scale mGWAS data as the exposure file and multiple myeloma GWAS data as the outcome file, to investigate the causal relationship between blood metabolites and multiple myeloma. This study also screens for relevant blood metabolites, and provides new ideas for early detection and treatment of multiple myeloma.

## Methods

### Data sources

Blood metabolite data were obtained from the Shin^17^ et al.'s mGWAS analysis study published in Nature Genetics in 2014, the largest genome-wide association study (mGWAS) of blood metabolites to date, pooling data from 7824 Europeans, including approximately 2.1 million single nucleotide polymorphisms, and 452 blood metabolites (GWAS ID: met-a). Multiple myeloma data from Burrows et al. 2021 Genome-wide association analysis data from UK Biobank, containing 372,617 samples (601 cases and 372,016 control) and 8.6 million single nucleotide polymorphisms (GWAS ID: ieu-b-4957).

### Conditions for SNP as an instrumental variable

①Instrumental variables were highly correlated with exposure, and the strength of the SNP was assessed using the *F *statistic, and if *F* > 10, the correlation between SNP and exposure was considered strong enough to insulate the results of the MR analysis from weak instrumental bias^15^. ②Instrumental variables were not directly correlated with outcome and only influenced outcome through exposure, i.e. no genetic pleiotropy was present, which was detected by MR-Egger regression in this study. ③ The instrumental variables are not related to confounding. The SNPs selected for the MR method should obey Mendel's law of genetics, i.e. parental alleles are randomly assigned to offspring and are not influenced by acquired factors such as socio-economic factors, and are therefore relatively independent and can theoretically be considered independent of confounding factors^18^.

### Selection of instrumental variables

Uniform criteria were set for SNP screening: ① *P* < 5 × 10^–8^ as statistically significant for inclusion in the study; ② linkage disequilibrium analysis with reference to the genotype of the European population (EUR) of the Thousand Genomes, which also needed to meet an LD threshold of r^2^ < 0.1 within 500 kb, retaining the single nucleotide polymorphism with the smallest *P *value.

### Statistical analyses for MR

Investigating the causal relationship between each blood metabolite and multiple myeloma by using the TwoSampleMR package (version 0.5.6) in the R^19^. In this study, inverse variance weighting (IVW) was used^20^ as the primary causal association effect assessment method. IVW is a method for MR to Meta-summarize the effects of multiple loci when analyzing multiple SNPs. IVW is used to ensure that all SNPs are valid instrumental variables and are completely independent of each other. In addition, we used the weighted median method (WME)^21^, MR-Egger regression^22^, simple mode-based estimation^23^ and weighted mode-based estimation^23^ to test the reliability and stability of the results. When the estimates of the causal association effects obtained from the above five different MR models were similar, we could conclude that the causal association between the metabolite and multiple myeloma was reliable and stable. If only one SNP remains, use the Wald ratio method. We also performed multiple hypothesis testing, using *P* < 1.10 × 10^–4^ (after Bonferroni correction) as the threshold indicating the presence of a direct causal association^24^ and 1.10 × 10^–4^ < *P* < 0.05 as a potential risk predictor for multiple myeloma. Heterogeneity tests and genetic pleiotropy tests were performed for causality for all metabolites with *P*_IVW_ < 0.05. At the same time, we calculated odds ratios as well as the power to detect a significant result across a range of odds ratios to help interpret the results and improve reproducibility^25^.

### Heterogeneity and sensitivity tests

Q-test for IVW and MR-Egger was used to detect potential violations of the assumption by the heterogeneity of the association between individual IVs. The included instrumental variables will be considered not heterogeneous when *P* > 0.05. The default fixed-effects model was employed if no substantial heterogeneity (*P* > 0.05) was observed; otherwise, the random-effects model was utilized^26^. MR-Egger was applied to estimate horizontal pleiotropy according to its intercept, ensuring that genetic variation was independently associated with exposure and outcome. When *P* > 0.05, it will be considered that there is less likely genetic pleiotropy in the causal analysis. This study used the leave-one-out method to assess the likelihood of associations observed by individual SNP driver.

### Metabolic pathway analysis

Following the MR analysis, we next used MetaboAnalyst5.0 software (https://www.metaboanalyst.ca/MetaboAnalyst/faces/home.xhtml)^27^ to perform metabolic pathway analysis.

### Ethics approval and consent to participate

No need for ethical approval as used of anonymous open data.

## Results

### Information on instrumental variables (SNP)

**Figure 1:**
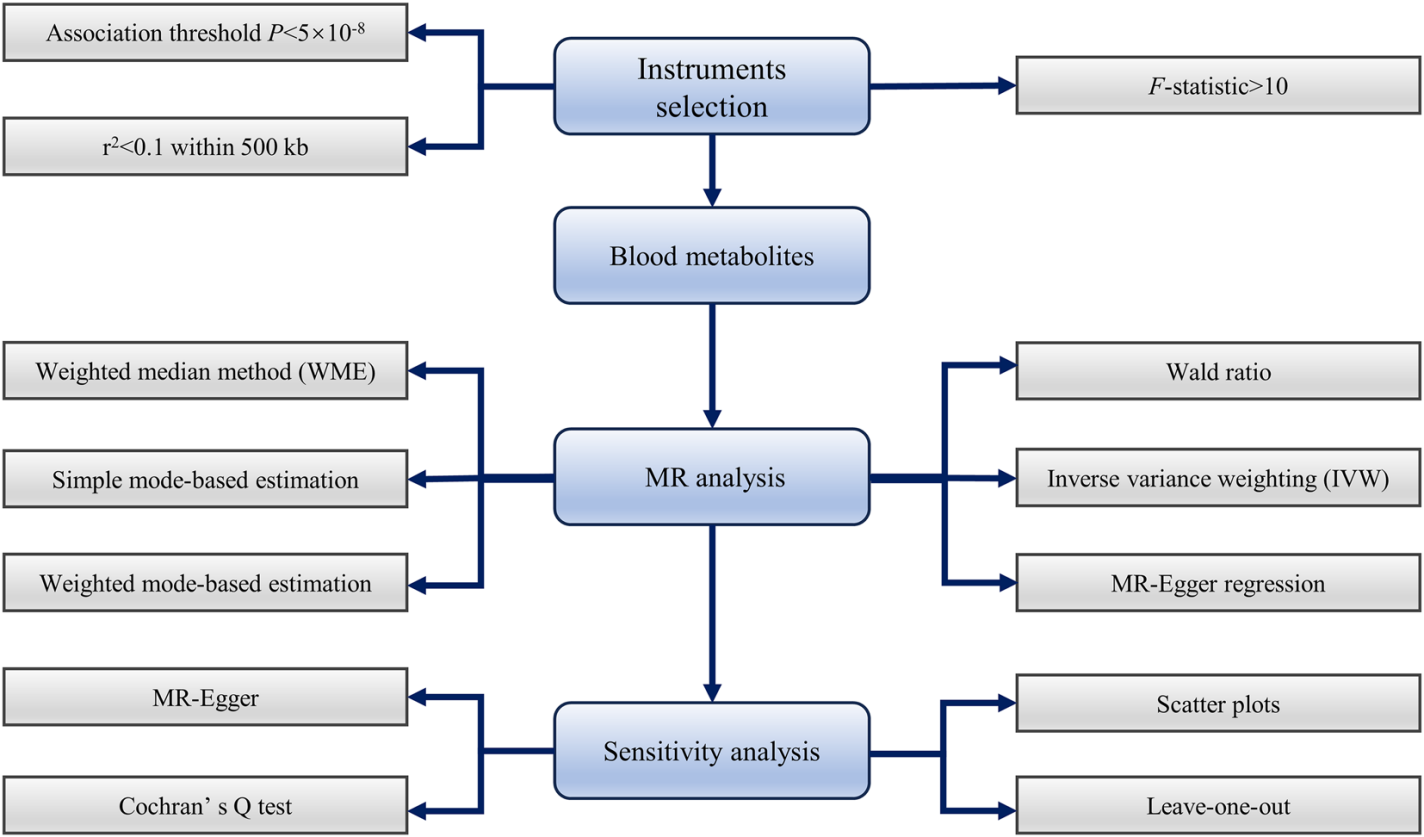
The flow chart of MR study revealing the causal relationship between human blood metabolites and the risk of MM. *MR* Mendelian randomization, *MM* multiple myeloma, *SNPs* single nucleotide polymorphisms.

Of all 2.1 million SNPs from 452 metabolites, we found 880 SNPs that met the selection criteria for instrumental variables, which was listed in Table S1. After overlapping these SNPs with multiple myeloma GWAS data, 839 SNPs were ultimately included in the follow-up analysis. These could be seen in Table S2. The flow chart of MR study is shown in Fig. 1. The minimum *F*-statistic for all SNPs included in the follow-up analysis was 28.81, indicating that the instrumental variables for metabolites were sufficiently plausible (*F* > 10).

### MR analyses results

**Figure 2:**
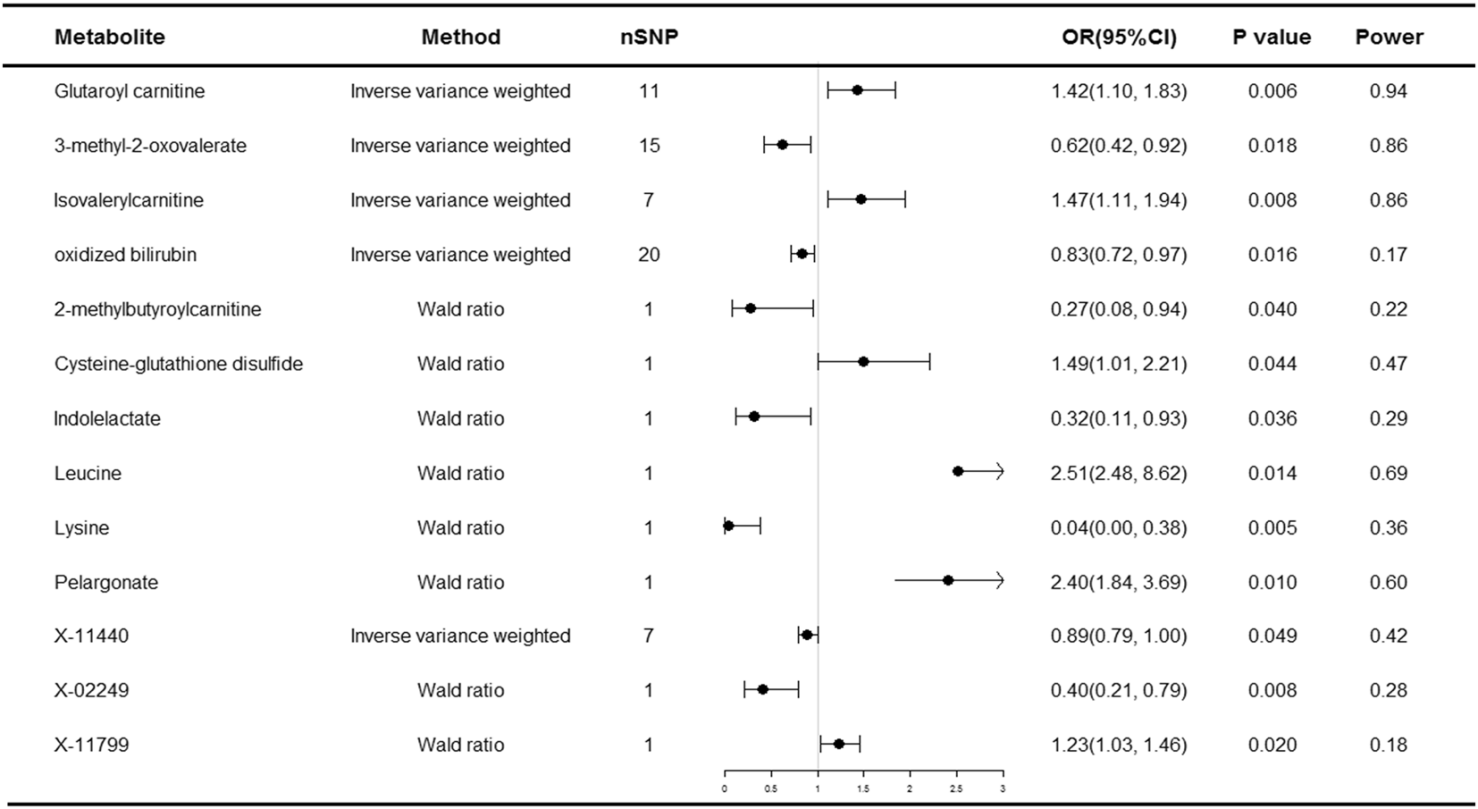
Forest plot for the causality of blood metabolites on multiple myeloma. *CI* confidence interval, *OR* odds ratio, *nSNP* number of single nucleotide polymorphism.

Of the 452 metabolites, thirteen metabolites were causally associated with multiple myeloma at a significant level (*P* < 0.05), including ten known metabolites and three unknown metabolites, as detailed in Fig. 2. After Bonferroni correction (*P* < 1.10 × 10^–4^), no metabolites were found that still had a significant effect.

Of the ten known metabolites, five may be associated with an increased risk of multiple myeloma: glutamine carnitine, isovalerylcarnitine, cysteine-glutathione disulfide, leucine and pelargonate; five may be associated with a decreased risk of multiple myeloma: 3-methyl-2-oxovalerate, oxidized bilirubin, 2-methylbutyroylcarnitine, indolelactate and lysine.

### Heterogeneity and sensitivity analysis

Four metabolites that reached statistical significance in IVW models were tested for heterogeneity and gene pleiotropy. P-values for heterogeneity and gene pleiotropy were greater than 0.05, indicating that none of the metabolites had heterogeneity or gene pleiotropy. The relevant results are shown in Table 1.

**Table 1:** Results of 5 MR models of known metabolites that reached statistical significance in IVW models and the heterogeneity and pleiotropy tests.

Metabolite	MR method	*P*	Heterogeneity	Horizontal pleiotropy
Glutaroyl carnitine	IVW	0.006	0.4842	
	MR Egger	0.885	0.4759	0.3619
	Simple mode	0.325		
	WME	0.082		
	Weighted mode	0.230		
3-methyl-2-oxovalerate	IVW	0.018	0.9996	
	MR Egger	0.293	0.9999	0.3952
	Simple mode	0.093		
	WME	0.025		
	Weighted mode	0.095		
Isovalerylcarnitine	IVW	0.008	0.9652	
	MR Egger	0.966	0.9458	0.6581
	Simple mode	0.060		
	WME	0.045		
	Weighted mode	0.074		
Oxidized bilirubin	IVW	0.016	0.5943	
	MR Egger	0.288	0.3979	0.8444
	Simple mode	0.146		
	WME	0.054		
	Weighted mode	0.034		

*MR* Mendelian randomization, *IVW* inverse variance weighting, *WME* weighted median method.

**Figure 3:**
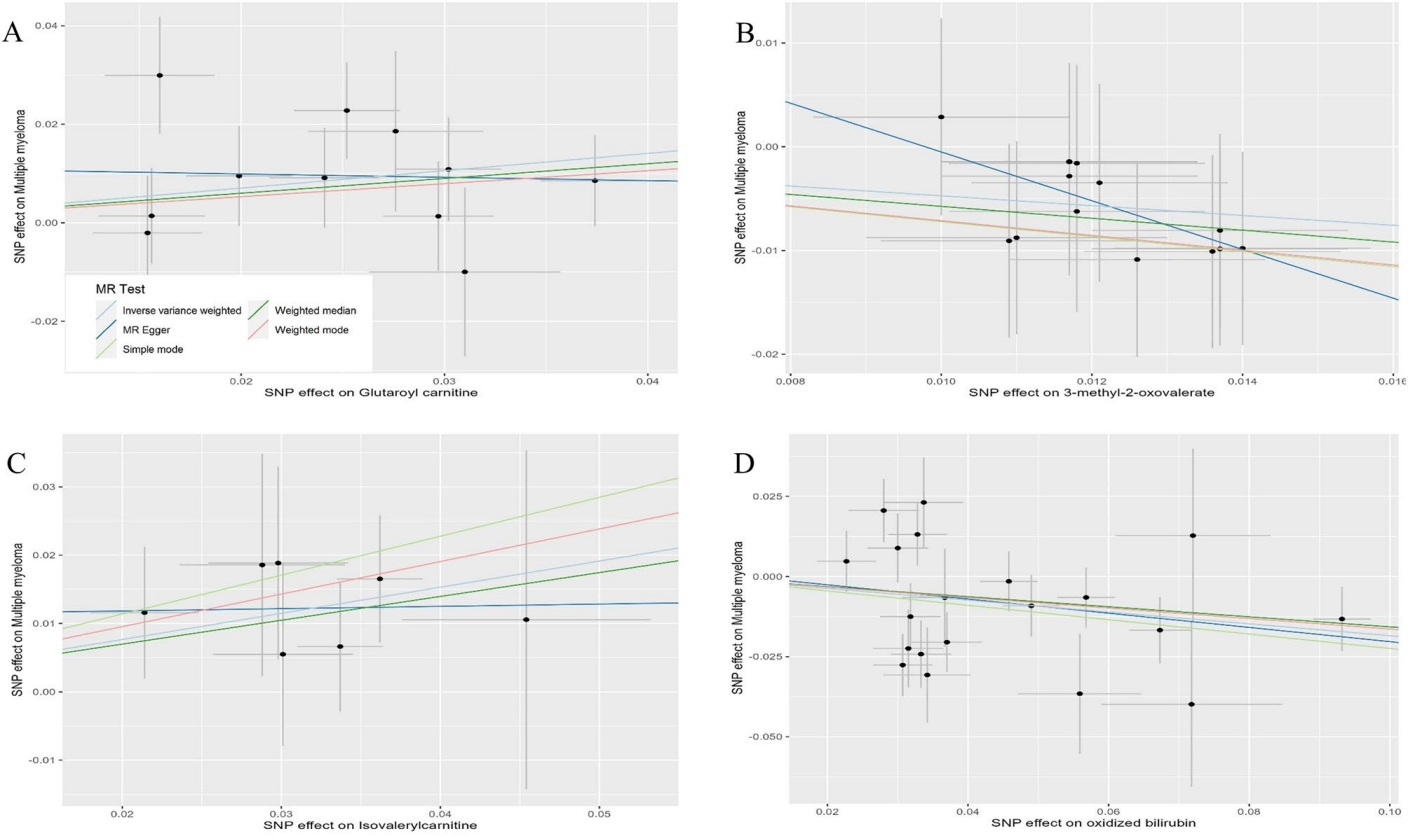
Scatter plots of the 5MR models for four metabolites with potential causal relationship with MM. **(A)** Glutamine carnitine, **(B)** 3-methyl-2-oxovalenate, **(C)** isovalerylcarnitine, **(D)** oxidized bilirubin. *MR* Mendelian randomization, *SNP* single nucleotide polymorphism.

Scatter plots of MR analysis results for the four metabolites are shown in Fig. 3.

**Figure 4:**
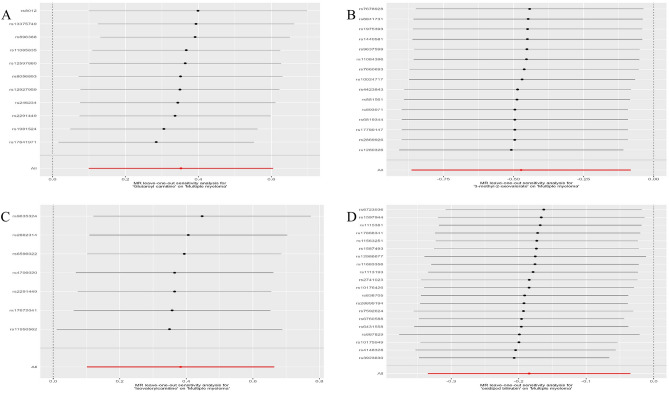
Leave-one-out forest map of four metabolites. **(A)** Glutamine carnitine, **(B)** 3-methyl-2-oxovalenate, **(C)** isovalerylcarnitine, **(D)** oxidized bilirubin. MR Mendelian randomization.

The sensitivity analysis of the above four metabolites by the leave-one-out method was robust, with no single nucleotide polymorphisms in any of the metabolites significantly affecting the results. Forest plots of the leave-one-out results for the metabolites are shown in Fig. 4.

### Metabolic pathway analysis

**Figure 5:**
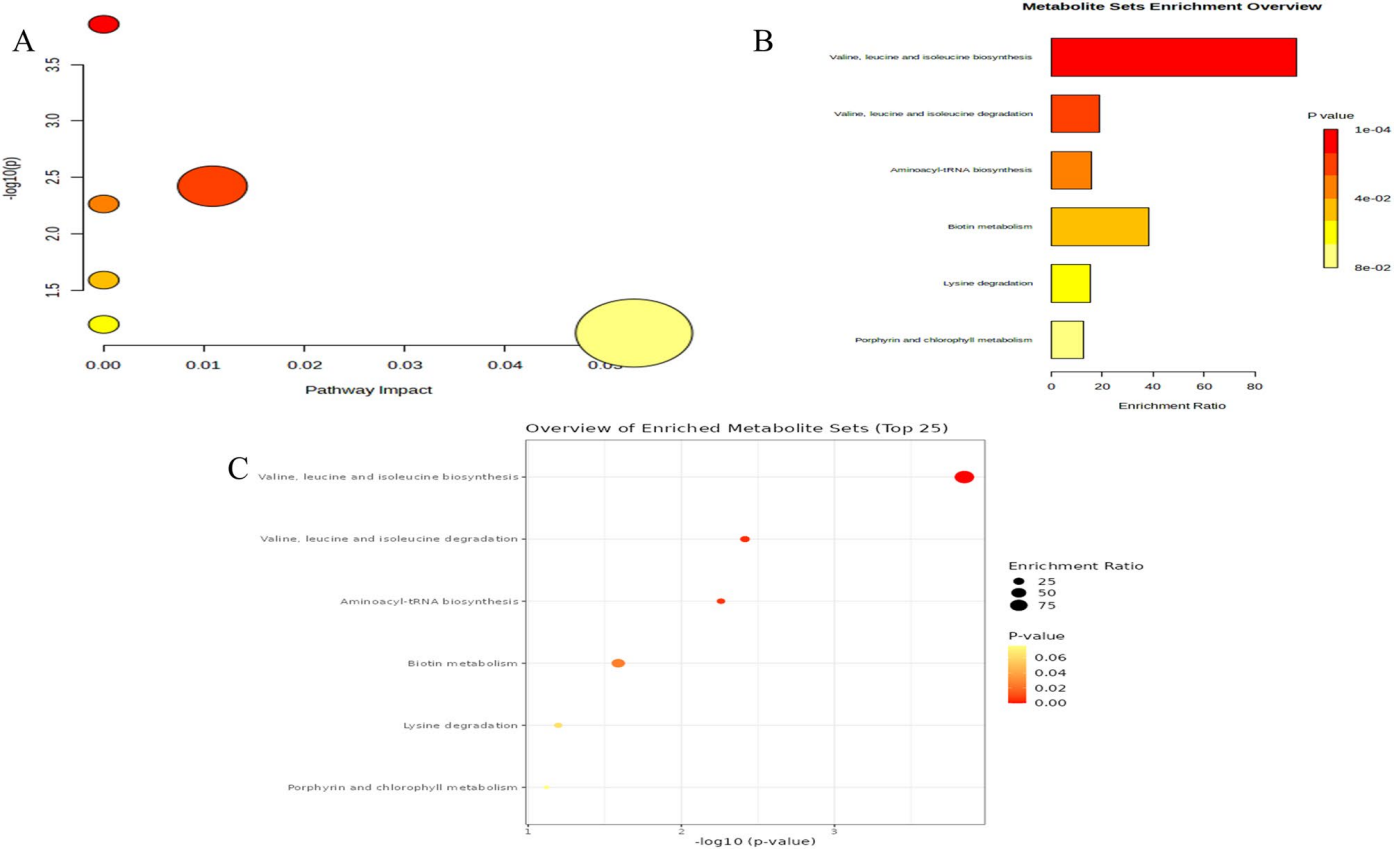
Metabolic pathway analysis and KEGG pathway enrichment analysis of ten metabolites by MetaboAnalyst5.0.

The ten metabolites screened were subjected to metabolic pathway analysis using MetaboAnalyst5.0 software and the results are shown in Fig. 5A. The metabolites were further subjected to KEGG pathway enrichment analysis and the results are shown in Fig. 5B, C. The metabolic pathway analysis showed that there were six metabolic pathways affecting multiple myeloma in the serum, four of which had statistically significant differences (*P* < 0.05), as shown in Table 2.

**Table 2:** Four metabolic pathways with statistically significant differences (*P* < 0.05).

Trait	Metabolic pathway	Involved metabolites	*P* value	Database
MM	Valine, leucine and isoleucine biosynthesis	3-Methyl-2-oxovaleric acid; leucine	1.42E−4	KEGG, SMP
MM	Valine, leucine and isoleucine degradation	3-Methyl-2-oxovaleric acid; leucine	3.84E−3	KEGG, SMP
MM	Aminoacyl-tRNA biosynthesis	Lysine; leucine	5.51E−3	KEGG
MM	Biotin metabolism	Lysine	2.58E−2	KEGG

## Discussion

Of the 452 blood metabolites involved in this study, ten are potential predictors of multiple myeloma risk. Five metabolites were included that may be associated with an increased risk of developing multiple myeloma, namely glutamine carnitine, isovalerylcarnitine, cysteine-glutathione disulfide, leucine and pelargonate. The other five may be associated with a decreased risk of multiple myeloma: 3-methyl-2-oxovalerate, oxidized bilirubin, 2-methylbutyroylcarnitine, indolelactate and lysine. Four of these metabolites, glutamine carnitine, 3-methyl-2-oxovalerate, isovalerylcarnitine and bilirubin oxide, reached statistical significance in IVW models.

The present study identified bilirubin oxide as a potential protective substance to reduce the risk of multiple myeloma. Li Volti et al.^28^ found that hematological malignancies exhibit an altered homeostasis of the redox balance, which can lead to the activation of various survival pathways that, in turn, lead to disease progression and chemoresistance. The heme oxygenase-1 (HO-1) pathway is thought to play an important role among these pathways. HO catalyzes enzymatic breakdown of heme, releasing carbon monoxide (CO), ferrous iron (Fe^2+^) and bilirubin oxide. As a degradation product of heme, bilirubin oxide inhibits the degradation of heme and thus the heme oxygenase-1 (HO-1) pathway. Raninga et al.^29^ also found that concurrent inhibition of HO-1 would improve therapeutic outcomes in MM patients.

Another metabolite that may reduce the risk of multiple myeloma is 3-methyl-2-oxovalerate, a branched-chain alpha-keto acid (BCKA) produced by the catabolism of isoleucine, which has been reported to be associated with type 2 diabetes and insulin resistance^30^. In a study on the catabolic pathway of branched-chain amino acids, 3-methyl-2-oxovalerate was the strongest predictor of IFG among the identified intermediate metabolites, independent of glucose^31^. Abnormal amino acid metabolism is one of the important features of MM. The important metabolic pathway of amino acids participates in protein synthesis as basic raw materials^32^. It has been shown that the metabolism of branched-chain amino acids influences the prognosis of multiple myeloma^33^. When catabolism of branched-chain amino acids is impaired, blood levels of 3-methyl-2-oxovalerate are reduced, potentially increasing the risk of multiple myeloma.

Glutamine carnitine and isovalerylcarnitine are both acylcarnitines^34^. Carnitine and acylcarnitine are key substances in cellular energy metabolism and can be synthesized from amino acids in the human kidney and liver. Their physiological roles include acting as sole carriers of long-chain fatty acids, transporting long-chain fatty acid classes into the mitochondria for beta-oxidation, and regulating the intracellular balance between free and acyl coenzyme A. Characteristic changes in one or more acylcarnitines indicate abnormal beta-oxidation of fatty acids and abnormal metabolism of branched-chain amino acids^35^. It has been shown that carnitine abnormalities are associated with metabolic diseases such as isoleucine and leucine metabolism disorders, isovaleric acidemia and type 2 diabetes^36^. It has also been suggested that carnitine can stimulate neuroprotective factors^37^. In the past study, isovalerylcarnitine has been proved to be able to activate the calpain system, producing an early and marked increase in apoptosis and cell killing^38^. MM cells were found to be dependent on glucose and glutamine metabolism in the first metabolic analysis of MM cells^39^. Higher levels of isoleucine and lower levels of glutamine and some lipids have been observed in myeloma patients at diagnosis, but not after remission^40^. Bajpai et al.^41^ were able to show that targeting the glutamine metabolism sensitizes MM cells to the bcl-2 inhibitor venetoclax. Studies of cellular metabolism have identified LDHA and HIF1α as novel targets for drug resistance in MM under hypoxic conditions in the bone marrow. Inhibition of LDHA and HIF1A can restore sensitivity to therapeutic drugs such as bortezomib. This suggests a correlation between branched-chain amino acid metabolism and the development of multiple myeloma, which is confirmed by metabolic pathway analysis.

This study is innovative in many ways: firstly, it combines metabolomics and genomics to investigate the causal relationship between blood metabolites as exposure factors and multiple myeloma using a Mendelian randomization approach, which has important clinical research value; secondly, this study uses multiple MR models and sets strict quality control conditions to make the results reliable and stable; finally, the large number of exposure factors involved in this study were metabolites in the blood, thus the analytical workload was huge and posed analytical challenges. There are also some limitations to this study. One limitation of our study is that the GWAS data we used were all from European populations, so generalization to other populations may be limited; secondly, although we identified a number of metabolites that were causally associated with multiple myeloma in our study, some of these were unknown metabolites and could not be studied for further analysis. Additionally, while MR analysis provides valuable insights into etiology, we must consider that blood metabolites can be influenced by various factors such as diet, host genetics, and the gut microbiome^42^. We can just conclude that blood metabolites are associated with multiple myeloma, but causation is not necessarily direct, thus it is important to note that our findings should be validated through rigorous RCTs and basic research before application in the clinic.

In summary, this study used a Mendelian randomization approach to explore the possible causal link between blood metabolites and multiple myeloma. Although no direct causal relationship was found, a number of potential risk predictors for multiple myeloma were identified, which has the potential to provide new insights into the influence of genetic-exposure interactions in the disease process of multiple myeloma. Furthermore, the analysis of both potential risk factors and associated metabolic pathways suggests that the metabolism of branched-chain amino acids may provide a new reference for the early screening and treatment of multiple myeloma.

### Supplementary Information

Supplementary Tables.

## Data Availability

The data and material that support the findings of this study are available in the IEU OpenGWAS, https://gwas.mrcieu.ac.uk/datasets/.
